# Accountable Care Organization Leader Perspectives on the Medicare Shared Savings Program: A Qualitative Study

**DOI:** 10.1001/jamahealthforum.2024.0126

**Published:** 2024-03-15

**Authors:** Dhruv Khullar, William L. Schpero, Lawrence P. Casalino, Reekarl Pierre, Samuel Carter, Yasin Civelek, Manyao Zhang, Amelia M. Bond

**Affiliations:** 1 Division of Health Policy and Economics, Department of Population Health Sciences, Weill Cornell Medical College, New York, New York; 2 Division of General Internal Medicine, Department of Medicine, Weill Cornell Medical College, New York, New York; 3 Center for Health Equity, Cornell University, New York, New York

## Abstract

**Importance:**

The Medicare Shared Savings Program (MSSP) includes more than 400 accountable care organizations (ACOs) and is among the largest and longest running value-based payment efforts in the US. However, given recent program reforms and other changes in the health care system, the experiences and perspectives of ACO leaders remain incompletely characterized.

**Objective:**

To understand the priorities, strategies, and challenges of ACO leaders in MSSP.

**Design, Setting, and Participants:**

In this qualitative study, interviews were conducted with leaders of 49 ACOs of differing sizes, leadership structures, and geographies from MSSP between September 29 and December 29, 2022. Participants were asked about their clinical and care management efforts; how they engaged frontline clinicians; the process by which they distributed shared savings and added or removed practices; and other factors that they believed influenced their success or failure in the program.

**Main Outcomes and Measures:**

Leader perspectives on major themes related to ACO initiatives, performance improvement, and the recruitment, engagement, and retention of clinicians.

**Results:**

Of the 49 ACOs interviewed, 34 were hospital-associated ACOs (69%), 35 were medium or large (>10 000 attributed beneficiaries) (71%), and 17 were rural (35%). The ACOs had a mean (SD) tenure of 8.1 (2.1) years in MSSP. Five major themes emerged: (1) ACO leaders reported a focus on annual wellness visits, coding practices, and care transitions; (2) leaders used both relationship-based and metrics-based strategies to promote clinician engagement; (3) ACOs generally distributed half or more of shared savings to participating practices; (4) ACO recruitment and retention efforts were increasingly influenced by market competition; and (5) some hospital-associated ACOs faced misaligned incentives.

**Conclusions and Relevance:**

In this study, the ACO leaders reported varied approaches to promoting clinician alignment with ACO goals, an emphasis on increasing annual wellness visits, and new pressures related to growth of other care models. Policymakers hoping to modify or expand the program may wish to incorporate these perspectives into future reforms.

## Key Points

**Question:**

A decade into the Medicare Shared Savings Program, what do leaders of accountable care organizations (ACOs) report as key priorities and challenges?

**Findings:**

In this qualitative study of 49 ACOs, leaders identified a focus on wellness visits, coding practices, and care transitions, and described using quality dashboards, personal relationships, and shared savings distributions to engage clinicians. Competition from Medicare Advantage presented a growing challenge for recruitment and retention of patients and practices, and some leaders of hospital-associated ACOs reported misaligned incentives.

**Meaning:**

Findings of this study suggest that policymakers may wish to consider the shifting strategies and challenges of ACO leaders as they reform the program.

## Teaser

This qualitative study assesses how leaders in accountable care organizations respond to the incentives of the Medicare Shared Savings Program.

## Introduction

Accountable care organizations (ACOs)—groups of clinicians and health care organizations that are financially responsible for the quality and costs of attributed patients—were first proposed in 2006 in response to a recognition that traditional Medicare payment did little to promote efficient and coordinated care and that previous incentive programs erroneously emphasized measurement at the individual instead of organizational level.^1^ Policymakers incorporated the concept into delivery system reforms of the Affordable Care Act, and hundreds of organizations have since participated in ACO models, the largest of which—the Medicare Shared Savings Program (MSSP)—has been in continuous operation for more than a decade. In 2022, approximately 11 million patients, or roughly 1 in 6 Medicare beneficiaries (and 1 in 3 of those in the fee-for-service program), were attributed to ACOs.^2^

Different ACO models and tracks have allowed organizations to assume varying levels of financial risk. In MSSP, an organization’s savings or losses are determined by comparing its annual costs relative to its own historical spending and since 2017 to regional spending benchmarks. Research suggests that MSSP has led to modest improvements in quality and spending for Medicare beneficiaries,^3,4,5,6^ but observers have also raised concerns about adverse patient selection, inadequate risk adjustment, and perverse benchmarking incentives.^7,8,9^

Prior survey-based and qualitative research, generally using data collected prior to 2018, has found that ACOs had introduced care plans to manage conditions in patients with medical complexity and engaged external management partners,^10,11^ and that ACOs were more likely than non-ACO organizations to use home visits and to provide clinician-level feedback to improve performance.^12,13^ In recent years, however, policymakers have introduced a number of changes to MSSP, and the current priorities, strategies, and challenges of ACO leaders remain incompletely characterized, especially in light of widespread changes in the US health care system, such as intensifying consolidation, growth of the Medicare Advantage program, and the COVID-19 pandemic. In this qualitative study, we aimed to understand how ACO leaders respond to the incentives of MSSP—a program that has matured into one of Medicare’s most prominent value-based payment efforts. Using in-depth semistructured interviews with a broad set of organizations, we examined the initiatives that ACO leaders prioritize to try to succeed in MSSP, their approach to clinician engagement and performance improvement, and the strategies they use to recruit and retain clinicians and practices.

## Methods

### Setting, Participants, and Study Design

We identified organizations that participated in MSSP using the 2022 Medicare Shared Savings Program ACO participant list.^14^ Because recruitment of organizations was conducted partly for a separate study on health care disparities, ACOs were eligible to be interviewed if at least 5% of their attributed beneficiaries were Black or dually eligible for Medicare and Medicaid, as determined by the Centers for Medicare & Medicaid Services (CMS) 2021 ACO Performance Year Financial and Quality Results file.^15^ To ensure adequate experience with the ACO program, we required organizations to have participated in MSSP for 4 or more years. These criteria resulted in a list of 229 eligible ACOs of the 483 organizations that participated in MSSP in 2022.

Similar to prior studies, we classified ACOs as “hospital-associated” if they included a hospital; otherwise, they were considered “physician-group” ACOs.^6^ We defined an ACO as rural if at least 25% of counties occupied by its practices were located in nonmetropolitan areas, as indicated by rural-urban commuting area codes.^16^ According to program rules, MSSP ACOs are required to have at least 5000 attributed beneficiaries, but no official definitions exist to categorize ACOs by size. In this study, we defined ACOs with fewer than 10 000 beneficiaries as small and those with 10 000 or more beneficiaries were considered medium or large.

We invited leaders of all 229 eligible ACOs to participate in the study by email (eAppendix 1 in Supplement 1). These individuals were sent 2 follow-up emails and received 2 follow-up phone calls if they did not respond to the initial invitation. If an ACO leader agreed to participate, they received a screening questionnaire to confirm how long the ACO had participated in MSSP, whether it included a hospital, and how many attributed beneficiaries it had in 2022.

In total, 49 ACOs agreed to participate. Between September 29 and December 29, 2022, 1 to 2 faculty members (D.K., W.L.S., L.P.C., or A.M.B.) conducted a 45-minute semistructured audio or video interview with 1 or more ACO leaders. Some leaders preferred to have other members of their team present; in these cases, interviews included 2 to 4 ACO participants. Interviews were conducted with individuals who held senior positions within ACOs, such as executive director or chief medical officer. These individuals did not receive compensation in exchange for being interviewed. Some participants preferred not to be taped. To ensure consistency across the interviews and to encourage interviewees to speak candidly, interviews were not recorded. Instead, an experienced research assistant (R.P. or S.C.) took detailed near-verbatim notes.

Because the study met exemption requirements, the Institutional Review Board at Weill Cornell Medicine waived review and informed consent. The study’s findings are presented in accordance with the Standards for Reporting Qualitative Research (SRQR) reporting guideline.

### Interview Protocol

The interview protocol (eAppendix 2 in Supplement 1) was developed through pilot interviews with ACO leaders and experts, as well as literature review of Medicare value-based payment programs. In addition to some specific questions about socially vulnerable patients for a separate study, the protocol included open-ended questions about the ACO key clinical and care management initiatives; how leadership decisions and strategies are communicated to clinicians; how shared savings are distributed; the process by which medical groups are added or removed; and other factors that leaders believe affect the likelihood of success or failure in the program.

### Data Analysis

The research team analyzed detailed notes taken by research assistants during the interviews. Major themes were identified using a framework analysis.^17^ Three research team members (D.K., R.P., and S.C.) began by inductively coding 10 interview notes. During this process, the researchers familiarized themselves with the interview notes, assigned labels to phrases or sentences that were striking, relevant, or repeated across multiple interviews, and then grouped related labels into broader categories of codes. This list of codes was then discussed with the entire research group, including a senior researcher with extensive qualitative research experience (L.P.C.), to develop a working analytic framework. The codes were iteratively refined as more interview notes were coded, and any differences of opinion among research team members were resolved through discussion, leading to consensus and a final set of codes. Finally, D.K. used this list to deductively code all interview notes using qualitative analysis software (ATLAS.ti, version 8.4.5; ATLAS.ti Scientific Software Development, GmBH), resulting in a final set of themes.

## Results

Of the 49 ACOs interviewed, 34 were hospital-associated (69%), 35 were medium or large (71%), and 17 were rural (35%); ACOs had a mean (SD) tenure of 8.1 (2.1) years in MSSP (Table 1). Participating ACOs had similar characteristics compared with nonparticipants (eTable in Supplement 1). Qualitative analysis of the interviews identified 5 major themes; these are presented in Table 2 along with representative quotations.

**Table 1.** Characteristics of 49 Participating ACOs

ACO characteristics	No. (%)
Size	
Small (<10 000 beneficiaries)	14 (29)
Medium or large (10 000 or more attributed beneficiaries)	35 (71)
Composition	
Hospital-associated	34 (69)
Physician-group	15 (31)
Geography	
Rural	17 (35)
Nonrural	32 (65)
Tenure	
Tenure in MSSP, mean (SD), y	8.1 (2.1)

Abbreviations: ACO, accountable care organization; MSSP, Medicare Shared Savings Program.

**Table 2.** Themes and Representative Quotations

Theme	Representative quotation
**Key programmatic initiative**	
ACO leaders reported several key initiatives, including increasing provision of annual wellness visits; ensuring that patients’ medical complexity was captured by rigorous coding efforts; and improving transitions of care, with a focus on postacute care.	“Our focus has been on annual wellness visits. We feel they help drive down ER visits, catch cancers, all that good stuff…We started embedding nurses in offices to do the heavy lifting.”
	“We’ve come to realize that Medicare wellness visits drive almost everything in terms of quality and cost.”
	“Accurately capturing the disease burden in our patient population is critical for success. It’s also become a bit of an arms race across the industry.”
**Clinician engagement**	
ACO leaders used both relationship-based and metrics-based strategies to promote clinician alignment with ACO goals.	“We generate very detailed reports that provide peer comparisons and actionable data. Everyone can see how they’re doing in general and how they compare to the doc down the hall.”
	“As a leader, I never tell clinicians, “Here’s what thou shalt do!”…It’s our role to understand the clinicians’ wish list and see how we can meet it.”
	“I personally visit every practice. I’m not some faceless bureaucrat.”
**Shared savings distribution**	
ACOs generally distribute half or more of shared savings to participating clinicians, usually at the practice or organization level, based on a combination of attribution, engagement, and quality performance.	“Distribution of savings depends on how badly you need to attract people. We have traditionally been able to keep 30% and distribute 70%. But in markets where there’s not much competition to get [practices] to join, it might be 50-50.”
	“Distributions are based primarily on number of attributed beneficiaries, but we’re developing systems to incentivize quality based on annual wellness visits, diabetes screening, appropriate coding, that sort of thing.”
	“We distribute based on quality metrics at the clinic level. We don’t want to do it at the physician-level or they’ll start playing “not it” with challenging patients.”
**Recruitment and retention**	
ACOs’ recruitment and retention efforts of practices and patients were increasingly influenced by market competition resulting from health system consolidation and large medical groups, as well as the growth of the Medicare Advantage program.	“We are being attacked by private equity, other health systems, other medical groups. We are desperate to stay alive, so we will take anyone who is willing to be engaged.”
	“Our problem is that Medicare Advantage has whittled away our numbers tremendously…I’m starting to feel that smaller ACOs just can’t compete with Medicare Advantage.”
	“In exchange for listening to our ACO spiel, we tell the [physicians] we’re giving them a ‘get-out-of-MIPS-free card.’”
**Hospital-associated ACOs**	
Leaders of hospital-associated ACOs held mixed views of whether hospitals help or hinder the ACO’s objectives, but many reported misaligned incentives.	“We’ve created a system of hospital-based systems whose goal is to fill hospital beds. They speak the right words…but all the actions are about putting resources into profitable cardiac and spine procedures.”
	“The hospitals are happy when Medicare patients are kept out of the hospital because they can fill their beds with commercial patients that generate more revenue.”
	“Compared to ACOs that don’t have hospitals, I think we’re at an advantage. We get timely data from our hospitals and it helps that we can talk to the people who run the inpatient case management teams.”
	“Nobody fights you on improving quality, but it’s hard to get alignment on reducing utilization…There might be an opportunity to reduce costs, but it’s not worth banging your head against the wall.”

Abbreviations: ACO, accountable care organization; ER, emergency room; MIPS, Merit-based Incentive Payment System.

### Theme 1: Key Programmatic Initiatives

ACOs engage in numerous clinical and care management activities, but many leaders reported recent efforts focused in 3 key domains: increasing provision of annual wellness visits; ensuring that patients' medical complexity was captured by rigorous coding efforts; and improving transitions of care, with a focus on postacute care. Increasing annual wellness visits was a major focus at many ACOs, with 1 respondent saying, “We’ve come to realize that Medicare wellness visits drive almost everything in terms of quality and cost.” Leaders described these visits as an opportunity to engage patients, to identify their needs, and to provide evidence-based preventive care.

Second, many ACO leaders reported that they had undertaken efforts to enhance coding practices to capture the clinical complexity of their organization’s attributed patients. This focus may have been accelerated by changes to MSSP financial benchmarking, including the introduction of regional benchmarking in 2017, through which an ACO’s per-beneficiary costs are compared with costs for other Medicare beneficiaries in the area. One leader said that coding has become “an arms race across the industry.” Prior research suggests that organizations that care for more socially disadvantaged populations exited MSSP at higher rates after the implementation of regional benchmarking, possibly because they were not able to fully capture the medical and social complexity of patients.^18^

A third major initiative centered on care transitions. Many ACO leaders reported investing heavily in care management programs that aim to improve the flow of patients across care settings through medication reconciliation efforts, posthospitalization phone calls, and early posthospitalization primary care visits. Leaders noted a special focus on managing postacute care—an area in which research has shown large practice variation—with many working closely with physicians to ensure that patients are referred to high-quality subacute rehabilitation and skilled nursing facilities.^19^

### Theme 2: Clinician Engagement

ACO leaders used both relationship-based and metrics-based strategies to promote clinician alignment with ACO goals. Many ACOs implemented dashboards for clinicians to view their performance on quality and cost measures; in some cases, these were unblinded to allow for peer comparisons. However, leaders reported that the extent to which clinicians choose to visit these dashboards—and to modify their behavior in response—remains variable.

In addition, ACO leaders emphasized the importance of personal relationships when it came to engaging clinicians in programmatic efforts. At some organizations, leaders regularly visited practices and met with clinicians to discuss the rationale for new initiatives and to jointly develop care delivery strategies. Although ACOs of all sizes reported relationship-based quality improvement efforts, the approach was most often emphasized by leaders of small ACOs. The leader of one small ACO indicated that the organization does not struggle with clinician engagement because “I personally visit every practice. I’m not some faceless bureaucrat.”

### Theme 3: Shared Savings Distribution

ACO leaders described a range of approaches to distribute savings when their organization met the minimum savings threshold, based on a combination of patient attribution, clinician engagement, and quality performance. Most leaders reported distributing at least 50% of savings to participating practices—often more in markets in which organizations faced greater competition for recruitment and retention. Most ACOs distributed savings to practices, not individuals, recognizing that many clinicians contribute to the care of a given patient. “We don’t want to do it at the physician-level or they’ll start playing ‘not it’ with challenging patients,” one leader said. Many leaders reported that payments were based on the number of attributed patients, although some reported trying to link payments to quality performance and clinician engagement. In general, practices could use savings as they saw fit; ACO leaders offered examples such as investing in new medical equipment, making bonus payments, and arranging staff appreciation events.

### Theme 4: Recruitment and Retention

ACOs’ recruitment and retention efforts of practices and patients were increasingly influenced by market competition resulting from health system consolidation and large medical groups, as well as the growth of the Medicare Advantage program. Some leaders reported difficulty maintaining interest and participation in MSSP, given the increasing numbers of patients and clinicians engaging in other forms of care. For example, between 2012 and 2022, the proportion of beneficiaries enrolled in Medicare Advantage plans relative to traditional Medicare nearly doubled, from 29% to 50%.^20^ At the same time, health system consolidation and private equity acquisition of physician practices have grown substantially, disrupting existing care patterns and networks.^21,22,23,24,25^

One leader said, “We are desperate to stay alive, so we will take anyone who is willing to be engaged.” Another noted difficulty retaining sufficient numbers of Medicare fee-for-service patients in the ACO, stating “Medicare Advantage has whittled away our numbers tremendously.” In general, leaders noted that practices were more likely to choose to leave the ACO than to be removed; some reported that practices were disaffiliated if they were minimally engaged with the ACO’s efforts or if they demonstrated consistently poor performance.

Several leaders said that a helpful recruiting tactic was contrasting ACO participation with the Merit-based Incentive Payment System (MIPS), which imposes administrative burdens on practices, often with minimal rewards.^26,27^ One leader reported that, “in exchange for listening to our ACO spiel, we tell the [physicians] we’re giving them a ‘get-out-of-MIPS-free card.’”

### Theme 5: Hospital-Associated ACOs

Leaders of hospital-associated ACOs held mixed views of whether hospitals help or hinder the ACO’s objectives, but many reported misaligned incentives. One leader said, “Nobody fights you on improving quality, but it’s hard to get alignment on reducing utilization.” Another reported that such alignment was possible, but primarily as a way to change the hospital system’s payer mix: “The hospitals are happy when Medicare patients are kept out of the hospital because they can fill their beds with commercial patients that generate more revenue.”

Other leaders, however, reported the inclusion of a hospital in the ACO could be an advantage. Hospitals often have more resources than medical groups to invest in quality-improvement processes, and integration with hospitals can aid in smoothing care transitions before and after hospitalization. One leader indicated that the ACO’s ability to communicate with inpatient care management teams was helpful, saying “It doesn’t mean that we get everything we want, but it is a lot easier than if we were just community-based [physicians] saying, “Hey, stop sending our patients to that [skilled nursing facility] with really poor quality!”

## Discussion

In interviews with 49 ACOs of varying sizes and structures across the US, we identified 5 major themes about the initiatives, challenges, and engagement strategies of ACOs participating in MSSP in 2022. Many leaders reported a growing focus on activities such as increasing annual wellness visits, enhancing coding practices, and improving transitions in care. Leaders described the use of quality dashboards and interpersonal relationships to engage clinicians in ACO programming, and most organizations distributed at least 50% of their shared savings to participants, usually at the practice level. In recent years, growing Medicare Advantage penetration, as well as private equity– and health system–based consolidation, presented difficulties for some ACOs in recruiting clinicians and retaining patients. Leaders of hospital-associated ACOs reported additional challenges related to conflicting incentives for reducing acute care utilization.

Our results suggest that greater research and policy attention is warranted in several areas. For example, most ACOs distributed shared savings at the practice level, not the individual clinician level, and future research could examine the outcomes of various distribution strategies with regard to clinician engagement and the quality of care at ACOs. In addition, our findings renew questions about whether aggressive coding practices, pursued by some ACOs, are an effort to heighten the risk profile of their attributed beneficiaries to secure favorable benchmarks and extract greater savings, as opposed to improving care quality or patient outcomes. Lawmakers have expressed ongoing concerns about the potential for upcoding in new ACO programs.^28^

Researchers should also continue to study the benefits and drawbacks of annual wellness visits for Medicare beneficiaries, which have increased substantially over time.^29,30^ Although some work suggests that these visits are associated with greater use of preventive services,^31,32,33^ they may also increase receipt of low-value care and their long-term implications remain uncertain.^34,35,36,37^ The Medicare Payment Advisory Commission (MedPAC) has raised concerns that wellness visits may allow for favorable selection in ACOs, especially those with retrospective patient attribution; a recent report found that Medicare beneficiaries in ACOs were more likely to receive these visits compared with non-ACO beneficiaries, and that ACOs provided a disproportionate share of the visits in the last quarter of the year, possibly in an effort to assign beneficiaries with low spending relative to claims-based risk scores.^9^ To mitigate this concern, MedPAC has recommended that all ACOs be required to adopt prospective beneficiary assignment.

Although MSSP remains a major value-based payment program in the US, Medicare now offers many options for clinicians to engage in value-based payment, and the Medicare Advantage program has grown substantially in the past decade. Some leaders reported that these and other trends have introduced difficulties in maintaining participation and engagement in MSSP. The implications of this shift are unclear: a decade after the implementation of ACOs, evidence of their effectiveness remains mixed relative to both traditional fee-for-service payment and the Medicare Advantage program.^38,39^ More research is needed to evaluate outcomes for patients attributed to ACOs vs those in other value-based payment programs, and CMS may also want to devote greater attention to facilitating multipayer ACO models.^40,41,42^

Early quantitative research found that physician-group ACOs were more likely to produce shared savings than hospital-associated ACOs, possibly because the latter face conflicting incentives when it comes to reducing acute care utilization; our study’s findings are consistent with this work.^6^ While there may be advantages for hospital participation (eg, greater capitalization, resources, and communication across care settings), leaders of hospital-associated ACOs described difficulties deploying programs that would reduce inpatient utilization. Some reported focusing instead on producing savings from postacute care and other types of care.

To our knowledge, no large-scale peer-reviewed qualitative study focused on MSSP ACO leadership, operations, and recruitment has been published in at least 5 years. Prior qualitative work on ACOs tended to focus on specific aspects of care, such as care coordination, home-based care, preventive services, and management of serious illnesses.^12,43,44,45^ A qualitative study on the first 18 months of the ACO program found that, at the time, leaders concentrated on transforming primary care and reducing avoidable emergency department use, and that “few ACOs were focused on any strategies around nursing homes or postacute care, such as improving care transitions between hospitals and postacute care facilities.”^43^ Our study suggests that, over the past decade, the latter has evolved into a primary concern for ACO leaders, and this finding is consistent with quantitative work showing that changes in postacute utilization account for a relatively large portion of the savings generated by ACOs.^46,47^

### Strengths and Limitations

A strength of this study is that it included a large number of interviews with ACO leaders. Although surveys can include an even larger and possibly more representative sample of ACO leaders, interviews can elicit more nuanced responses from ACO leaders and can discover perspectives not foreseen when creating a survey (or interview) protocol. However, this study also has limitations. First, we could not determine whether the reports of ACO leaders were associated with objective improvements in care; future work should aim to link qualitative and quantitative analyses to understand which aspects of ACO operations, culture, and leadership are connected to better quality or lower costs. Second, it is possible that interviewees selectively presented favorable parts of their work and that a different set of questions, such as those focused on program failures, might have resulted in a different range of responses. This concern may be somewhat mitigated because participants were assured that their responses would be anonymized and aggregated. Third, the interview protocol included a focus on care for socially vulnerable patients for a separate study; however, the interviews also included a broad set of questions regarding global ACO management and care delivery, and participants were provided ample opportunity to discuss these areas. Fourth, although this study included leaders from a broad range of ACOs and is among the largest of its kind, the perspectives of these individuals may not generalize to other organizations, especially newer ACOs and those that care for very few low-income individuals or members of racial and ethnic minority groups.

## Conclusions

In this study of 49 ACOs of varying sizes and structures across the US, 5 major themes about the initiatives, challenges, and engagement strategies of ACOs participating in MSSP in 2022 were identified. Accountable care organizations maintain bipartisan consensus and Congress appears committed to continuing the model, which is the only Medicare value-based payment program authorized by statute. As CMS continues to refine the ACO program and introduce new opportunities for clinicians and health care organizations to engage in value-based payment, it may benefit from an understanding of the priorities, strategies, and challenges of ACO leaders, as well as how the incentives embedded in national policy manifest on the front lines of care delivery.

## Notes

Supplement 1. **eAppendix 1.** Invitation Email **eAppendix 2.** Interview Protocol **eTable.** Characteristics of Participating Versus Non-Participating ACOs

Supplement 2.
Data Sharing Statement
