# Supplementary material for: Accountable Care Organization Leader Perspectives on the Medicare Shared Savings Program: A Qualitative Study
Source: JAMA Health Forum. 2024 Mar 15;5(3):e240126. doi: 10.1001/jamahealthforum.2024.0126 (PMC10943415; doi:10.1001/jamahealthforum.2024.0126)
Supplement: Supplement 1. — eAppendix 1. Invitation Email eAppendix 2. Interview Protocol eTable. Characteristics of Participating Versus Non-Participating ACOs [file jamahealthforum-e240126-s001.pdf]

Supplemental Online Content

Khullar D, Schpero WL, Casalino LP, et al. Accountable care organization leader perspectives on the Medicare Shared Savings Program: a qualitative study. *JAMA Health Forum.* 2024;5(3):e240126. doi:10.1001/jamahealthforum.2024.0126

eAppendix 1. Invitation Email

eAppendix 2. Interview Protocol

eTable. Characteristics of Participating Versus Non-Participating ACOs

This supplemental material has been provided by the authors to give readers additional information about their work.

© 2024 Khullar D et al. *JAMA Health Forum.*

eAppendix 1. Invitation Email

Subject: Interview Request for Cornell Research on ACOs

Dear [Invitee],

We are studying the strategies and programs that ACOs use to improve care for their Medicare patients as a whole and for socially vulnerable populations in particular.

We would very much appreciate it if you can find the time to participate in a telephone interview to share your insights on this topic.

We will use interviews to inform our research, but at no time will we identify you or your organization, verbally or in the articles we publish.

Our team has previously published numerous articles in the *New England Journal of Medicine*, *JAMA*, and other medical and health policy journals. The findings from this study will be submitted for publication in a peer-reviewed journal. The research is funded by two non-profit, non-partisan organizations, Arnold Ventures and the Physicians Foundation.

We will be happy to email you a copy of any articles that result from this study.

If you are interested in participating, please respond to this message (or contact us by phone at XXX-XXX-XXXX) and we can answer any questions you may have. A member of our research team can then help schedule a 45-minute interview at your convenience. Prior to the interview, we will send you a list of topics we are hoping to explore.

Sincerely,

XX

© 2024 Khullar D et al. *JAMA Health Forum*.

eAppendix 2. Interview Protocol

Description of Project

Hello, my name is [Interviewer Name] from Weill Cornell Medicine. I'm on the line with my colleague [research coordinator], who will be taking notes. As we mentioned when we first contacted you, we would like to understand the strategies and programs used by your ACO to improve care for patients.

This project is jointly funded by Arnold Ventures and the Physicians Foundation, but neither organization was involved in the development of the questions. Please know that your responses will be de-identified and anonymous. We will not reveal your name or the name of your organization at any point. If you like, we can e-mail you a copy of any articles we publish.

We are interested in what programs and care improvement initiatives your ACO has undertaken, and which populations they target, with a particular focus on socially vulnerable populations, such as low-income individuals and members of racial/ethnic minority groups.

Questions

1. What do you think has been the most effective program your ACO uses to improve the health of your attributed patients during the past few years?

   Probes [ask if not spontaneously discussed by the interviewee]:

   a) Could you tell us a bit more about this program?
      a. When did the program start?
      b. Has the program changed over time?
   b) Why do you think this program works so well?
   c) Do you think the program works well for both Medicare patients in general and for socially vulnerable patients in particular?
      a. Why or why not?

2. Has your ACO introduced any other major care improvement programs or wrap-around services (e.g., social work, transportation, housing support, financial assistance, mental health resources, etc.) during the past few years?

   Probes [ask if not spontaneously discussed by the interviewee]:

© 2024 Khullar D et al. *JAMA Health Forum*.

a) Could you tell us a bit more about this program?
    a. When did the program start?
    b. Has the program changed over time?

b) How well do you think the program(s) work?

c) Do you think the program works well for both Medicare patients in general and for socially vulnerable patients in particular?

Why or why not?

3. Do any of your programs focus on specific patient populations (e.g., low-income patients, communities of color, medically complex patients, or certain clinical conditions)?

   Probes [ask if not spontaneously discussed by the interviewee]:

   a) Could you tell us a bit more about this program?
      a. When did the program start?
      b. Has the program changed over time?

4. What are the key challenges you face in improving care for socially vulnerable populations?

   a. Are there payment and/or other policy changes that Medicare or other payers could make that would enhance your organizations' ability to care for socially vulnerable populations?

5. Do you partner with other community organizations, such as behavioral health programs, substance use programs, home health agencies, or federally qualified health centers?

6. How are decisions about new programs or changes to existing programs made within the ACO?

   a. Who are the key decisionmakers?
   b. How are decisions about new strategies and programs communicated to medical groups and frontline clinicians?

7. What are the key factors that you consider when thinking of adding new medical groups or discontinuing affiliation with existing ones?

8. Does your ACO routinely screen for Socioeconomic Determinants of Health (SDOH)?

© 2024 Khullar D et al. *JAMA Health Forum.*

a) if so: what tool do you use?
b) what are the advantages and disadvantages of screening?

9. In what ways do you make use of data to inform care delivery within the ACO?

10. Are shared savings distributed in any way?

11. [If the ACO includes one or more hospitals]:

    a) What are ways in which the hospital helps (or hinders) your efforts to improve your ACO's care for socially vulnerable patients?

12. [If the ACO is operated by Aledade or some other third party]:

    a) What are ways in which [Aledade or whichever organization] helps (or hinders) your efforts to improve your ACO's care for socially vulnerable patients?

13. Is there anything else you would like to tell us? Anything we should be asking about but didn't?

© 2024 Khullar D et al. *JAMA Health Forum.*

eTable. Characteristics of Participating versus Non-Participating ACOs

ACO Characteristics	49 Participating ACOs (N or %)	180 Non-Participating ACOs (N or %)
**Size**		
- Small (<10,000 beneficiaries)	14	61
- Medium/Large (≥10,000 beneficiaries)	35	119
**Composition**[a]		
- Hospital-integrated	34	-
- Physician-group	15	-
**Geography**[b]		
- Rural	17	-
- Non-rural	32	-
**Tenure**		
- Mean tenure in MSSP	8.1 years	7.6 years
**Attributed Beneficiary Demographics**[c]		
- % dual-eligible	8.7%	8.0%
- % American Indian/Alaska Native	0.2%	0.2%
- % Asian/Pacific Islander	2.1%	2.1%
- % Black	10.4%	10.4%
- % Hispanic	1.8%	1.8%
- % White	82.0%	81.7%
- % Other	3.8%	3.8%

Abbreviations: ACO, Accountable Care Organization; MSSP, Medicare Shared Savings Program. [a,b] ACO composition and geography were not able to be ascertained for non-participants. [c] Beneficiary demographics are based on analysis of 2021 ACO performance year financial and quality results data.

© 2024 Khullar D et al. *JAMA Health Forum*.
