# Supplementary material for: Accountable Care Organization Leader Perspectives on the Medicare Shared Savings Program: A Qualitative Study
Source: JAMA Health Forum. 2024 Mar 15;5(3):e240126. doi: 10.1001/jamahealthforum.2024.0126 (PMC10943415; doi:10.1001/jamahealthforum.2024.0126)
Supplement: Supplement 2. — Data Sharing Statement [file jamahealthforum-e240126-s002.pdf]

Data Sharing Statement

Khullar. Accountable Care Organization Leader Perspectives on the Medicare Shared Savings Program. *JAMA Health Forum*. Published March 15, 2024.
doi:10.1001/jamahealthforum.2024.0126

Data
Data available: No

Additional Information
Explanation for why data not available: Data for this study was conducted through semi-structured interviews and participants were assured that all responses would be confidential and anonymous.
